# Trends, projection and inequalities in full immunization coverage in Ethiopia: in the period 2000-2019

**DOI:** 10.1186/s12887-022-03250-0

**Published:** 2022-04-11

**Authors:** Kalkidan Yibeltal, Sitota Tsegaye, Hasset Zelealem, Walelegn Worku, Meaza Demissie, Alemayehu Worku, Yemane Berhane

**Affiliations:** 1 Department Reproductive Health and Population, Addis Continental Institute of Public Health, Addis Ababa, Ethiopia; 2 Department of Nutrition and Behavioral Sciences, Addis Continental Institute of Public Health, Addis Ababa, Ethiopia; 3 Department of Epidemiology, Georgia State University School of Public Health, Atlanta, USA; 4 Department of Global health and Health Policy, Addis Continental Institute of Public Health, Addis Ababa, Ethiopia; 5 School of Public Health, College of Health Sciences, Addis Ababa University, Addis Ababa, Ethiopia; 6 Department of Epidemiology and Biostatistics, Addis Continental Institute of Public Health, Addis Ababa, Ethiopia

**Keywords:** Full immunization coverage, Trend, Projection, Inequality, Demographic and health survey and Ethiopia

## Abstract

**Background:**

Immunization is among the most cost-effective health interventions to improve child survival. However, many countries in sub-Saharan Africa failed to achieve their national and international coverage targets repeatedly. The present study investigated trends of coverage and inequalities in coverage in Ethiopia.

**Methods:**

This study used data from five rounds of the Demographic and Health Surveys conducted in Ethiopia in 2000, 2005, 2011, 2016, and 2019. The surveys used a multistage cluster sampling procedure to obtain a nationally and sub-nationally representative data. The outcome variables included in the study were full immunization coverage and inequality. The World Health Organization’s Health Equity Assessment Toolkit was used to conduct the inequality analysis. Projections for 2025 were based on smoothed averages generated using the demographic and health survey data from 2000 to 2019.

**Results:**

The full (basic) immunization coverage in Ethiopia has increased steadily from 14.3% in 2000 to 44.1% in 2019. Based on the average past performance, the immunization coverage is projected to reach 53.6% by 2025, which will be short of the 75% national full (basic) immunization coverage target for the year 2025. Mothers with higher levels of education are more likely to get their children all basic vaccinations than those with lower levels of education. Similarly, the inequality gaps due to wealth and residency are significant; where children in the lowest wealth strata and those living in rural areas remained disadvantaged.

**Conclusion:**

Despite a steady increase in immunization coverage in the past two decades the country is yet to achieve its immunization target. Thus, more efforts are needed to achieve the current and future national immunization targets. A more focused intervention targeting the disadvantaged groups could be an effective strategy to achieve coverage and minimize the inequality gaps in immunization.

## Background

Immunization program is among the most cost-effective health interventions that helped to significantly improve child survival globally [1]. The success of eradication of smallpox in 1980 after more than a decade long campaign by the World Health Organization (WHO) laid the foundation for the initiation of the Expanded program on Immunization (EPI) [2, 3]. Since the introduction of the EPI by the WHO in 1974, immunization have reduced child deaths and morbidity radically globally and notably in low-income countries [1, 4]. In Ethiopia, the EPI was launched in 1980 with an ambitious goal of achieving universal childhood immunization coverage of children under 2 years of age by 1990 [5]. Later, in 1986, the goal was confined to children under 1 year of age to reach a coverage of 75% by 1990 [5, 6].

Despite the steady increments in immunization coverage over the past decades; pro-urban, pro-rich, pro-educated inequities in the coverage have been observed in Sub-Saharan African (SSA) countries [7, 8]. The persistent low full immunization coverage in low-and middle-income countries (LMICs) is attributed to weak health system to deliver services, high dropouts, shortage of supplies, vaccine stock out, cold chain breakages, and lack of immunization culture [7, 9–12]. Studies have shown that children from the urban areas have better immunization coverage than their rural counterparts [4, 13, 14] which could be attributed to better access to health facilities, better provision of childhood immunization services, and better attitude and knowledge of the mothers regarding immunization [12, 15]. In addition to place of residence, children from neighborhoods with high poverty, illiteracy and unemployment rates were more likely to be unimmunized [8]. A study based on secondary analysis of Demographic and Health Surveys (DHSs) and Multiple Indicator Cluster Surveys (MICS) also indicated that place of residence, maternal education, socioeconomic status and birth order are the most common factors associated with lower immunization coverage [16]. A WHO report on the state of inequality in childhood immunization showed higher inequalities by mothers’ education level and household economic status in low-income countries where full immunization coverage was at least 20 percentage points higher in the richest wealth quantile compared to the poorest quantile [17]. However, such inequalities are not precisely captured and used in program planning due to persistent discrepancies in reports on immunization coverage from different sources of data within a country. In Ethiopia immunization coverage discrepancies between DHS, national EPI survey, administrative estimates, and annual WHO/UNICEF estimates of National Immunization Coverage (WUENIC) are commonly observed [18].

Different strategies have been used to increase uptake of immunization including reaching every district (RED) [19], Sustainable Outreach Services (SOS) [5]**, i**ntegrating door-to-door immunization strategy with the fixed point facility immunization [20]; SMS vaccination reminder [21, 22] and reminder sticker [23]. Despite these continuous efforts, Ethiopia is still lagging behind the set target on many occasions. Even though there are many studies that have assessed immunization coverage [4, 6, 24, 25], studies that showed coverages combined with projections and inequalities in coverage among different population groups are scanty. This study provides evidence that guides public health actions to improve coverage and equity in access and utilization of childhood immunization services. The objective of this study was to assess trends in full childhood immunization coverage and trends in inequalities in coverage in Ethiopia.

## Method

### Study setting

Ethiopia is located in the horn of Africa. It is the second most populous country in Africa next to Nigeria and the twelfth most populous country in the world with a total population of more than 114.9 million people in 2020 [26]. Administratively, during the study periods, Ethiopia was divided into nine regional states and two city administrations. The country follows a three-tier health care delivery system in which the EPI is implemented at all levels of the tier. Besides the static sites, the immunization services are also provided as an outreach and by mobile teams. In all service delivery points immunization is given voluntarily and free of charge [5].

### Study design

Secondary data from multiple demographic and health cross-sectional surveys conducted in Ethiopia in five rounds from 2000 to 2019 was used for this study. The coverage trends and inequalities in immunization coverage were assessed based on household wealth status, mother’s educational status and place of residence.

### Study population

The respondents to the survey were mothers (aged 15-49) with live children in the age group of 12-23 months and who were permanent residents of the selected household or those who spent the night before each survey in the selected households.

### Sampling procedure

The stratified multi-stage sampling strategy used in the DHSs in Ethiopia is globally standardized and applied in many LMICs [27]. Briefly, the DHS sampling procedure allows selecting a nationally and sub-nationally representative sample based on the population size of each sub-nation (region) in the country.

The sampling frame used for each DHS in Ethiopia was based on the Ethiopian population and housing census conducted prior to the surveys [28–33]. The sampling frame contains information about the Enumeration Area (EA) location, type of residence (urban or rural), and the estimated number of residential households. EAs were selected using probability sampling proportional to size of the region and each region was stratified in to urban and rural areas. A household listing operation was carried out in all of the selected EAs. The resulting lists of households served as a sampling frame for the selection of households in each EA, also referred as cluster. A fixed number of households per EA were selected based on the newly created household listing. All women age 15-49; having 12-23 months of age child; and who were either permanent residents of the selected households or visitors who stayed in the household the night before the survey were eligible for the surveys [28–32].

### Data collection

The DHS collects data using a structured questionnaire that has several modules. For this study, data collected using household questionnaire, woman’s questionnaire and health facility questionnaire were used. The vaccination status of the eligible children was compiled from the mothers’ interview, vaccination card and health facility record.

The data were accessed by requesting the DHS program website [34] after submitting the purpose of the study. We obtained the data for the years 2000, 2005, 2011 and 2016 from DHS. The mini-DHS 2019 full dataset is not yet publicly available.

### Data analysis

The outcome variables were full immunization and inequality whereas household wealth index, mother’s educational status and place of residence were the independent variables.

A child is considered to be fully immunized when he/she had received one dose of Bacille Calmette-Guérin (BCG) vaccine, three doses of polio vaccine, three doses of the combined diphtheria, tetanus toxoid and pertussis (DTP3) vaccine, and one dose of measles vaccine [30]. Each vaccine antigen variable was coded as “0” for those who didn’t receive the vaccine dose and “1” for those who have received the dose. Then these values were added to give the immunization status. The immunization status was recoded as “1” (full immunization) if the child had received all the recommended doses of the vaccines mentioned above and “0” (incomplete immunization) if the child has missed at least one of the vaccines doses mentioned above.

Health inequalities are observable differences in health indicators between subgroups of a population. Subgroups can be defined by geographic or socioeconomic factors such as economic status, education and place of residence [35].

The full immunization coverage was calculated by dividing the number of children aged 12–23 months receiving full/basic immunization (one dose of BCG, three doses of polio, three doses of DTP3, and one dose of measles) by the total number of children aged 12–23 months participated in the survey.

DHS is a very broad survey that is conducted in multiple countries and cover plenty of indicators with a detailed design and methods; any data analysis using data from DHS need to consider that complexity. Thus, we used a complex data analysis statistical technique that takes in to consideration the nature of the data using “svy” command which is a Stata command for fits statistical models for complex survey data. It adjusts the results of a command for survey settings identified by svyset. Due to the non-proportional allocation of the sample to different regions and their urban and rural areas we need to ensure that the results are representative at the national and regional levels. Thus, sampling weights must be used to analyze the EDHS data. Since the DHS uses a two-stage stratified cluster sample, sampling weights were calculated based on sampling probabilities separately for each sampling stage and for each cluster in order to compensate for the unequal probability of selection between the different regions and their urban and rural areas as well as for non-response. A thorough explanation of the weighting procedure is found in the DHS reports [28–32]. Projections for 2025 were made using a smoothed average using the data from DHS during 2000-2019. The smoothed average was established based on the average coverage reported in each DHS cycle and then weighted for the period 2000 to 2019. For this study, an annual increment in coverage was calculated by dividing the difference of the two consecutive survey coverage by the interval between the two surveys using the following formulas:

$$\mathrm{Annual}\ \mathrm{increment}\ 1\left(\mathrm{from}\ 2000-2005\right)=\frac{Full\ immunization\ coverage\ 2005-2000}{5}$$

$$\mathrm{Annual}\ \mathrm{increment}\ 2\ \left(\mathrm{from}\ 2005-2011\right)=\frac{Full\ immunization\ coverage\ 2011-2005}{6}$$

$$\mathrm{Annual}\ \mathrm{increment}\ 3\ \left(\mathrm{from}\ 2011-2016\right)=\frac{Full\ immunization\ coverage\ 2016-2011}{5}$$

$$\mathrm{Annual}\ \mathrm{increment}\ 4\ \left(\mathrm{from}\ 2016-2019\right)=\frac{Full\ immunization\ coverage\ 2019-2016}{3}$$

The projected national full immunization coverage for the year 2025 was calculated by adding the mean smoothed average for the period 2000-2019 multiplied by 6 on to the 2019 coverage, the calculation is shown below:

$$=\left(2019\ DHS\ coverage\right)+\left( mean\ for\ 2000-2019\ast 6\right)$$

$$=44.1+\left(1.58\ast 6\right)=53.6$$

Where mean is the smoothed average of the yearly increment in full immunization coverage from 2000 to 2019.

Six is the year difference between the last Ethiopian DHS in the year 2019 and the target year 2025.

The primary objective of the Ethiopia national EPI comprehensive multi-year plan (2016-2020) was to achieve at least 90% national coverage and 80% in every district with all vaccines by 202 0[5]. This target is currently adjusted to 75% to be achieved by 2025 according to the Ethiopian Health Sector Transformation Plan II (2020/21-2024/25) [36].

The inequality analysis was conducted using the WHO Health Equity Analysis Toolkit (HEAT) software**,** Version 4.0 (beta) Geneva, World Health Organization,2020 [37]**.** Summary measures used to assess inequalities were relative concentration index and ratio. The ratio is a simple measure of inequality which do not account for the population share and measures the ratio of two population subgroups. Since place of residence has only two categories (urban and rural) in order to measure the relative inequality, ratio was used as a summary measure. The ratio is the immunization outcomes in the most-advantaged (urban) group divided by the immunization outcomes in the most-disadvantaged group (rural). The ratio takes a value of one if there is no inequality. For favorable health indicators like full immunization coverage, values greater than one indicates a concentration among the advantaged (urban) and values smaller than one indicates concentration among the disadvantaged subgroup (rural) [38].

Since educational status and household wealth index have more than two subgroups with ordered dimensions, the relative concentration index was used as a summary measure to assess the inequality among the subgroups. The relative concentration index (RCI) is a complex measure of inequality which shows the health gradient across population subgroups by taking into account all population subgroups on a relative scale. The value of RCI is bounded between − 100 and 100 and takes the value of 0 when there is no inequality. Positive values indicate a concentration of the indicator among the advantaged (richest, secondary and higher education), while negative values indicate a concentration of the indicator among the disadvantaged (poorest, no education). The greater the absolute value of RCI, the higher the level of inequality [38].

In order to add the 2019 data, we reconstructed the concentration curves and concentration indices for the years 2000-2019 using the XY (scatter) chart-type in Microsoft Excel. The inequality analysis was done for household wealth quintiles (lowest or poorest, second, middle, fourth, highest or richest), mother’s education status (no education, primary, secondary and above), and place of residence (rural/urban).

The concentration index “*Conc-I*” was computed using the following formula:

$$Conc-I=\left(p1L2-p2L1\right)+\left(p2L3-p3L2\right)+\dots +\left(p\mathrm{T}-1L\mathrm{T}-p\mathrm{T}L\mathrm{T}-1\right)$$

where *p* is the cumulative proportion of children, *L* is the cumulative proportion of fully immunized children, and T is the number of socioeconomic groups.

When the concentration index increases or when the concentration curve moves away from the line of equality it shows greater inequality in distribution of the health variable of interest (full immunization), on the other hand, when the concentration curve gets closer to the line of equality it shows a lesser inequality in the distribution of the health variable.

## Results

### Full immunization coverage trend (2000-2019) and projections for 2025

**Fig. 1:**
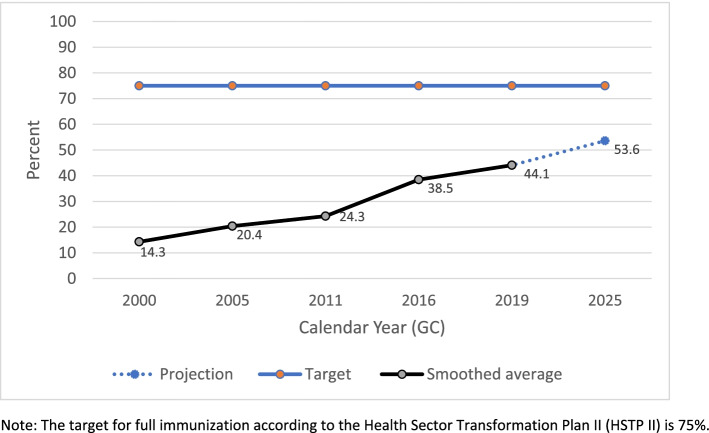
National full immunization coverage trend 2000-2019 and projections for 2025 based on smoothed average for Ethiopia. Note: The target for full immunization according to the Health Sector Transformation Plan II (HSTP II) is 75%

**Fig. 2:**
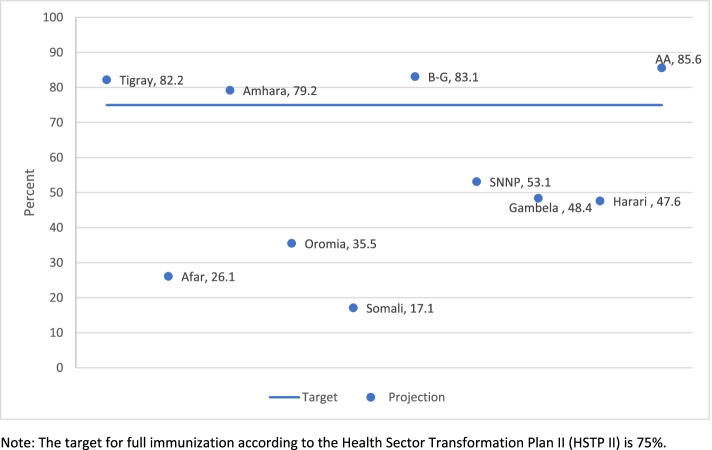
Regional/sub-national full immunization coverage projected for 2025 compared to target for Ethiopia. Note: The target for full immunization according to the Health Sector Transformation Plan II (HSTP II) is 75%

The full immunization coverage in Ethiopia has been increasing steadily from 14.3% in 2000 to 44.1% in 2019 through each survey year (Table 1). The projection based on the smoothed average showed the coverage will reach 53.6% by 2025 which will be far below the target 75% set for 2025 (Fig. 1). According to the projection for 2025 based on the smoothed average yearly increment, only 4 of the 11 regions namely Tigray (82.2%), Benishangul-Gumuz (82.2%), Addis Ababa (85.6%) and Amhara (79.2%) will achieve the 75% target (Fig. 2). On the other hand, Somali (17.1%), Afar (26.1%) and Oromia (35.5%) will have the lowest coverage for full immunization by 2025 based on the smoothed average projection (Fig. 2).

**Table 1-:** Ethiopian National full immunization coverage trend 2000-2019

Year	National Full immunization Coverage	95% CI
2000	14.3%	12.1 - 16.4%
2005	20.4%	17.3 - 23.4%
2011	24.3%	20.9- 27.7%
2016	38.5%	34.3 - 42.8%
2019	44.1%	37.2 - 51.0%

### Inequality analysis

**Fig. 3:**
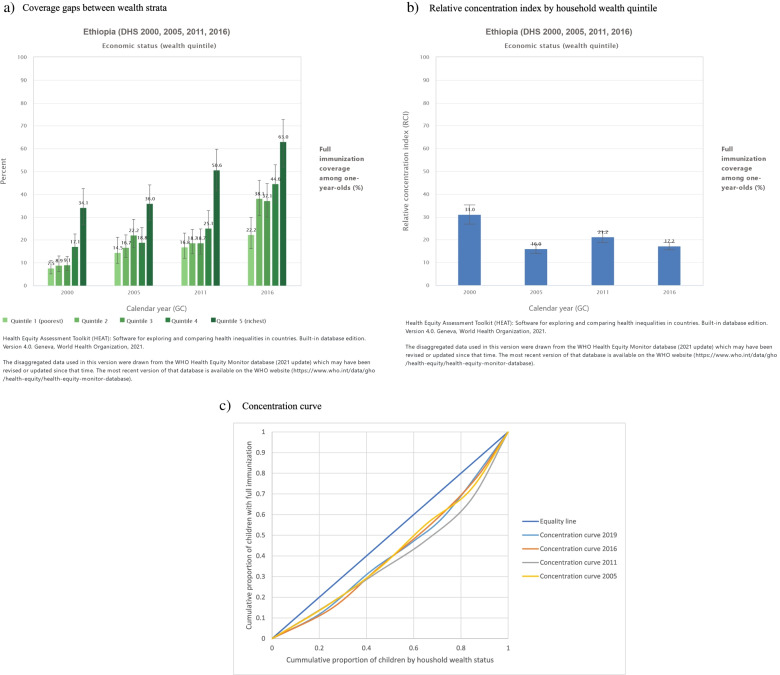
Full Immunization coverage inequality among wealth groups in Ethiopia, 2000-2016. **a** Coverage gaps between strata; **b** Relative concentration index by household wealth quintile and **c** Concentration curve

The inequality analysis by wealth quintile revealed that the poorest are lagging behind where coverage showed consistent progressive increment among the better-off group since 2000 (Fig. 3a). Even though the wealth inequality gaps showed a decreasing tendency since 2005 the reduction was neither consistent nor significant as indicated in the relative concentration index (Fig. 3b). The concentration curve also showed that there has been a reduction in inequality by wealth status in 2019 compared to the gap in 2005 (Fig. 3c) whereas the inequality gap by wealth in 2011 was the greatest as shown in Fig. 3c where the concentration index is 0.2203.

**Fig. 4:**
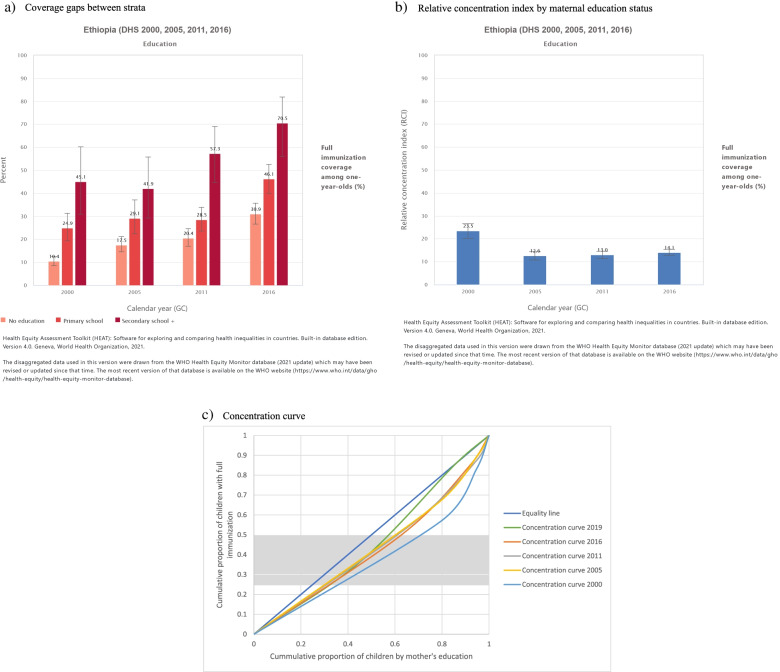
Full Immunization coverage inequality by maternal education status in Ethiopia. 2000-2016. **a** Coverage gaps between strata **b** Relative concentration **c** Concentration curve

The inequality analysis by educational status showed that children of uneducated mothers had the lowest immunization coverage in all the surveys (Fig. 4a). As shown in Fig. 4b, there had been no significant reduction in inequality based on mother’s education status from 2000 to 2016 (Fig. 4a). However, according to the concentration curve, the inequality gap seemed to narrow in 2019 where the concentration index for the year 2019 was 0.0774 compared to 0.2382 for the year 2000 (Fig. 4c).

**Fig. 5:**
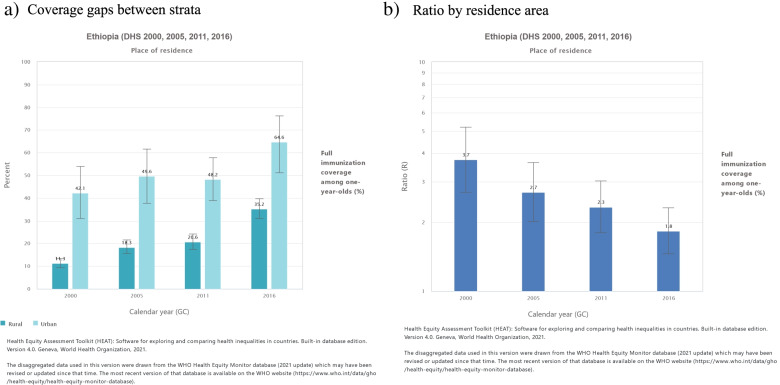
Full Immunization coverage inequality by residence area (rural Vs urban) in Ethiopia. 2000-2016. **a** Coverage gaps between strata and **b** Urban-rural inequality ratio

The immunization coverage had been consistently better in urban areas though steady improvements in coverage were observed in the rural areas (Fig. 5a). The rural-urban inequality gap appeared to be closing mainly due to steady improvements in rural areas as shown in Fig. 5b.

## Discussion

Our study revealed that the national full immunization coverage had steadily increased during 2000-2019 but failed to reach targets for 2020 [9] and unlikely to reach the national target by 2025 if program performance continues at the projected smoothed average level. There were significant inequality gaps by household wealth, mother’s education and place of residence favoring the most advantaged households [39].

The national full (basic) immunization coverage had shown a similar overall increment in the last couple of decades as observed in many other African countries [40–42]. The increments were attributed to improved immunization planning at national and subnational levels, strengthening financial resources from domestic and external sources, and improvements in community level health service delivery [5, 43]. The implementation of reaching every district using Quality Improvement (RED-QI) in Ethiopia has also shown to increase the immunization coverage and immunological response [19]. Despite the immediate improvements seen in the immunization program after implementing different strategies technical as well as financial challenges restricted their success in the long run. Thus, many LMICs including Ethiopia are not able to achieve their immunization coverage targets at national and sub-national levels [44].

Inequality among regions/states is also a well know phenomenon in the immunization program across Africa. Our analysis showed only four regions (Tigray, Addis Ababa, Benishangul gumuz and Amhara) will likely reach the national target by 2025 based on our projections. Some of the regions with the lowest figures based on the projection like Afar and Somalia have hard to reach areas with populations predominantly pastoralist [24]. Those areas may require customized strategies including tracking population movements and settlements, engaging clan leaders, strengthening cross-border collaborations, mapping water points and livestock markets, developing pastoralist-focused communication materials, launching joint human-animal vaccination campaigns, and establishing permanent transit vaccination points [45].

The observed inequalities in full immunization coverage based on wealth quintile were similar to previously reported studies [14, 46–49]. Even though vaccination services are provided free of service charges at the public health facilities, the observed inequalities by wealth status are likely to emanate from the costs incurred for transportation and loss of productivity [46, 50]. Another important social factor for low uptake of immunization is the low social status of women which negatively affects access to vaccination because of their limited decision-making power to even seek health services for their childre n[50]. Despite the establishment of increasing number of service delivery points closer to the community in the last two decades, the inequality due to wealth status had persisted in Ethiopia [46, 48].

This study revealed that educated mothers vaccinate their children better than uneducated mothers. This finding also concurs with other studies in LMICs [46, 49, 51]. These findings can be related to better awareness about the importance of vaccination among mothers with higher level education. Educated women could also have greater autonomy to choose health care services that generate better health for themselves and their children [52]. Education enables mothers to have better health seeking practices with an increased uptake of both curative and preventive services such as immunization [53]. In addition, it is likely that educated mothers to be partner with educated men which play an important role in seeking health care and immunization services [54].

The full immunization coverage in children residing in the urban areas is much higher than those residing in rural areas [4, 15]. The rural-urban inequality can be explained by the degree of accessibility of health facilities and awareness about immunization [24]. However, efforts to mitigate accessibility through intensive community mobilization and expanding basic health services via community based health program in rural areas has helped to narrow the inequality gaps [5]. Similar efforts have shown improvements in basic health service utilization in the rural areas in other LMICs [42, 55].

It is known that the immunization programs are highly vulnerable to unexpected public health emergencies as well as social, political and economic crisis, either at subnational, national and/or global levels. Such natural and manmade emergencies can hinder immunization programs efforts by disrupting the normal function of nations and can potentially derail past gains. The Coronavirus disease 2019 (COVID-19) pandemic preventive measures including physical distancing and travel restrictions [56] have posed serious challenges to uptake of preventive child health service [57, 58]. The negative effect of the pandemic on the immunization program has already been noticed in Africa [59]. The pandemic might further pull back the performance of the immunization as a result of resource mobilization, staff displacement, low health seeking behavior of the public due to fear of getting the infection by visiting health facilities.

Some limitations worth mentioning include the quality of the vaccination data, which was collected from the vaccination cards when available and by maternal recall. As significant proportion of the data was obtained from maternal recall, thus a recall bias is highly likely. Maternal report has poor agreement with coverage data obtained from facility-based records and serology [60]. Since DHS measure wealth status in relative term, we were not able to directly compare findings across countries or over time [61]. The trend analysis was also made based on data collected in approximately 5 years interval. We calculated the annual smoothed average based on the available data but that may not actually reflect the annual performance level. The smoothed average could also be affected by the performance at the beginning and/or at end of the period. However, as there are more data quality issues in the annual coverages reported by the program, it is still believed the trend analysis fairly represents the overall performance of the immunization program at the national and sub-national levels in Ethiopia. Another limitation is as the study is descriptive it has not taken into consideration confounders and multicollinearity issues.

## Conclusion

The national immunization coverage has been growing steadily but not at a rate enough to meet past and future national childhood full immunization targets. The inequality gaps may continue to undermine program efforts to achieve universal coverage unless addressed in a sustained manner and using all locally appropriate strategies. Aiming interventions on the disadvantaged groups could be an effective strategy and could reduce morbidity and mortality due to vaccine preventable diseases. Reducing the observed inequalities in full immunization require focused policies and strategies at both national and subnational levels.

## Data Availability

The datasets generated and/or analyzed during the current study are available at https://dhsprogram.com/data/available-datasets.cfm

## Abbreviations

AA: Addis Ababa
BCG: Bacillus Calmette–Guérin
B-G: Benishangul-Gumuz
CSA: Central Statistical Agency
COVID-19: Coronavirus disease 2019
DPT: Diphtheria, Pertussis (whooping cough), and Tetanus
EA: Enumeration area
EDHS: Ethiopia Demographic and Health Survey
EPI: Expanded Program on Immunization
GC: Gregorian Calendar
HEAT: Health Equity Assessment Toolkit
HepB: Hepatitis B
Hib: *Haemophilus influenzae* type b
HEW: Health Extension workers
IRB: Institutional Review Board
LMICs: Low-and Middle Income Countries
MICS: Multiple Indicator Cluster Survey
PHC: Population and Housing Census
RED: Reaching Every District
SMS: Short Message Service
SNNP: Southern Nations Nationalities and People
SSA: Sub-Saharan Africa
UCI: Universal Child Immunization
WHO: World Health Organization
